# Evaluation of a community-based intervention package to improve knowledge of obstetric danger signs, birth preparedness, and institutional delivery care utilization in Arba Minch Zuria District, Ethiopia: a cluster-randomized trial

**DOI:** 10.1186/s12978-023-01713-w

**Published:** 2023-11-18

**Authors:** Mekdes Kondale Gurara, Veerle Draulans, Yves Jacquemyn, Jean-Pierre Van Geertruyden

**Affiliations:** 1 Department of Public Health, College of Medicine and Health Sciences, Arba Minch University, Arba Minch, Ethiopia; 2 Global Health Institute, Faculty of Medicine and Health Sciences, University of Antwerp, Wilrijk, Belgium; 3 Faculty of Social Sciences, Centre for Sociological Research, KU Leuven, Louvain, Belgium; 4 Department of Obstetrics and Gynaecology, Antwerp University Hospital, UZA, Edegem, Belgium

**Keywords:** Birth preparedness, Community-based intervention, Institutional birth rate, Maternal health, Skilled attendance at birth

## Abstract

**Introduction:**

Maternal healthcare utilization, particularly the institutional delivery, is disproportionately low in rural Ethiopia. This study aimed to evaluate the effectiveness of an integrated package of community-based interventions on the improved knowledge of obstetric danger signs, birth preparedness, and institutional delivery services utilization in rural areas of Gamo zone, southern Ethiopia.

**Methods:**

We conducted cluster-randomized controlled trial (NCT05385380) from 2019 to 2021 at the Arba Minch Health and Demographic Surveillance System site. We randomly assigned the 10 kebele clusters to intervention and control arm. We used a package of interventions, which included providing information on safe motherhood via video and/or audio with a birth preparedness card for pregnant women, training for community volunteers and health extension workers, and improving maternity waiting home services. Women in the control arm received routine services only. We used generalized mixed-effects logistic regression models to evaluate the effectiveness of the intervention on the outcome variables.

**Results:**

The study enrolled 727 pregnant women across the 10 clusters, with a 617 (84.9%) successful follow-up rate. The proportion of institutional delivery in the intervention arm was increased by 16.1% from 36.4% (174/478) at the baseline to 52.5% (224/427) at the endline (Adjusted odds ratio [AOR] for McNemar’s Test = 1.5; 95% confidence interval [CI]: 1.1 to 2; p < 0.001). In the control arm, however, there was a 10.3% fall in the proportion of institutional delivery (from 164/249 to 105/190). Pregnant women who received the intervention were significantly more likely to give birth in a health institution than those who did not (AOR 2.8; 95% CI: 1.2, 6.4).

**Conclusion:**

The study demonstrates that an integrated community-based intervention package that included video-based storytelling and upgrading maternity waiting homes increased institutional delivery care utilization among rural women. We recommend that audio-visual storytelling, starting during pregnancy and continuing postpartum, be incorporated into routine maternal healthcare services to address access to care inequalities in rural settings.

*Trial registration: *The study protocol was registered in the clinicaltrials.gov with registry number NCT05385380.

## Abstract (plain language summary)

Many women in developing nations, including Ethiopia, are dying due to problems related to pregnancy and childbirth. One of the interventions to prevent maternal illness and deaths is promoting and ensuring the timely use of maternal health care services. This study aimed to evaluate the effectiveness of an integrated package of community-based interventions on the improved institutional birth rate in rural Ethiopia. We conducted a trial at the Arba Minch HDSS site. The package provided information on safe motherhood via videos and audiocassettes for pregnant women, a birth preparedness card for women, community volunteers and extension workers training, and maternity waiting home services upgrading. In the control arm, women received routine services only. From the 10 Arba Minch HDSS kebele clusters, six kebele clusters were randomly assigned to the intervention and an additional four were assigned to the control. Different statistical techniques were used to evaluate the effectiveness of the intervention on the institutional birth rate. At the baseline, 727 pregnant women had enrolled across all 10 clusters, with a 617 (84.9%) successful follow-up rate. The intervention arm had a higher proportion of institutional birth (224/427 [52.5%]) at the endline than the baseline (174/478 [36.4%). Furthermore, the study showed a significant association between intervention status and institutional birth rate. Therefore, stimulating demand for existing services to ensure the timely use of care can improve maternal health service utilization, particularly the institutional birth rate.

## Background

The third United Nations’ Sustainable Development Goal (SDG) aims to reduce the global maternal mortality ratio (MMR) from 216 per 100,000 live births in 2015 to less than 70 per 100,000 by 2030. As a member state of the United Nations, Ethiopia is working towards achieving SDG targets. The MMR in Ethiopia is estimated at 412 per 100,000 live births in 2020 [1]. The MMR is significantly higher in rural areas of Ethiopia including the less-developed districts of the rural Gamo zone [2, 3]. Most maternal deaths are caused by direct obstetric complications, some of which cannot be predicted, but can be successfully prevented or treated if and only if timely access to emergency obstetric care (EmOC) and skilled attendance are accessed [4].

Despite the Ethiopian government's efforts to increase the number of health facilities through its health extension programme (HEP), access to EmOC continues to be a challenge, and the met need for EmOC in 2016 was low (18% in all facilities) [5, 6]. Most rural women encounter difficulties using existing EmOC services because of limited availability; furthermore, a lack of knowledge about pregnancy complications and the resulting lack of understanding of the importance of timely access to care facilities hamper the use of EmOC. Poor infrastructure and limited resources compounded by difficult terrain and the sparse distribution of rural populations are the main reasons for the low utilization of EmOC facilities in Ethiopia [7].

For this and other reasons, women residing in rural and geographically remote communities continue to give birth outside health institutions in unsafe and unhygienic conditions [5, 7–9]. To improve access to EmOC, the Ethiopian government has implemented various strategies, including the use of maternity waiting homes (MWHs) [10]. MWHs are residential lodgings located near health facilities where females can stay during pregnancy to enable timely access to EmOC [11] and was promoted by the government as part of improving access to EmOC, particularly in remote and rural settings [12]. Accordingly, most basic EmOC facilities in Ethiopia have constructed MWHs [6], and in 2015, a standardized health facility guideline for the implementation of MWHs was approved by the Federal Ministry of Health [13].

Regarding MWH contributions to maternal health, several studies from LICs, including Ethiopia, have reported that MWHs improve the institutional birth rate and reduce the odds of homebirth [14–18]. However, pooled evidence of studies conducted up to 2021 in Ethiopia still shows low (44.9%) utilization of MWHs [19]. A study conducted in the Gamo zone of southern Ethiopia in 2019 showed that only 8.4% of pregnant women used MWHs [20], 9.7% completed the continuum of care [21] and only one-quarter of births were assisted by skilled attendants [21]. These studies also reported a low level of awareness about the benefit of MWHs, poor quality of care at MWHs, the absence of food catering at the MWHs and high costs associated with transportation as factors associated with low utilization [22].

A previous study from the study setting also revealed that only a few rural women gave birth in health institutions, despite government efforts to address the barriers to accessing institutional delivery care services [5]. Another study from the same area found that the magnitude of the first, second, and third maternal delays was high, with delay one being the most common due to unfamiliarity with obstetric danger signs, a lack of birth preparedness, poor perceptions of facility care, and geographic barriers [23].

A lack of knowledge about the importance of maternal health services, including MWHs, is believed to negatively influence demand for these services. This demand is embedded and intertwined with cultural and social practices [24]. Hence, a strategy of promoting maternal health services is crucial to increase demand for and access to EmOC facilities [19]. In addition, women are more likely to use services if they are of high quality [25]. Studies have shown that individual interventions that focus on limited factors to improve institutional delivery care utilization have been largely ineffective [26–28]. However, scientific evidence on the effect of an integrated community-based intervention package during pregnancy for improving institutional delivery among pregnant women is scarce. Therefore, this study aimed to examine the effectiveness of a community-based package of interventions, including improving pregnant women's knowledge on danger signs, improving risk perceptions through video-based storytelling, improving birth preparedness and complication readiness practices, and upgrading MWHs, increase the institutional delivery service utilization in rural Ethiopia.

## Materials and methods

### Setting

**Fig. 1:**
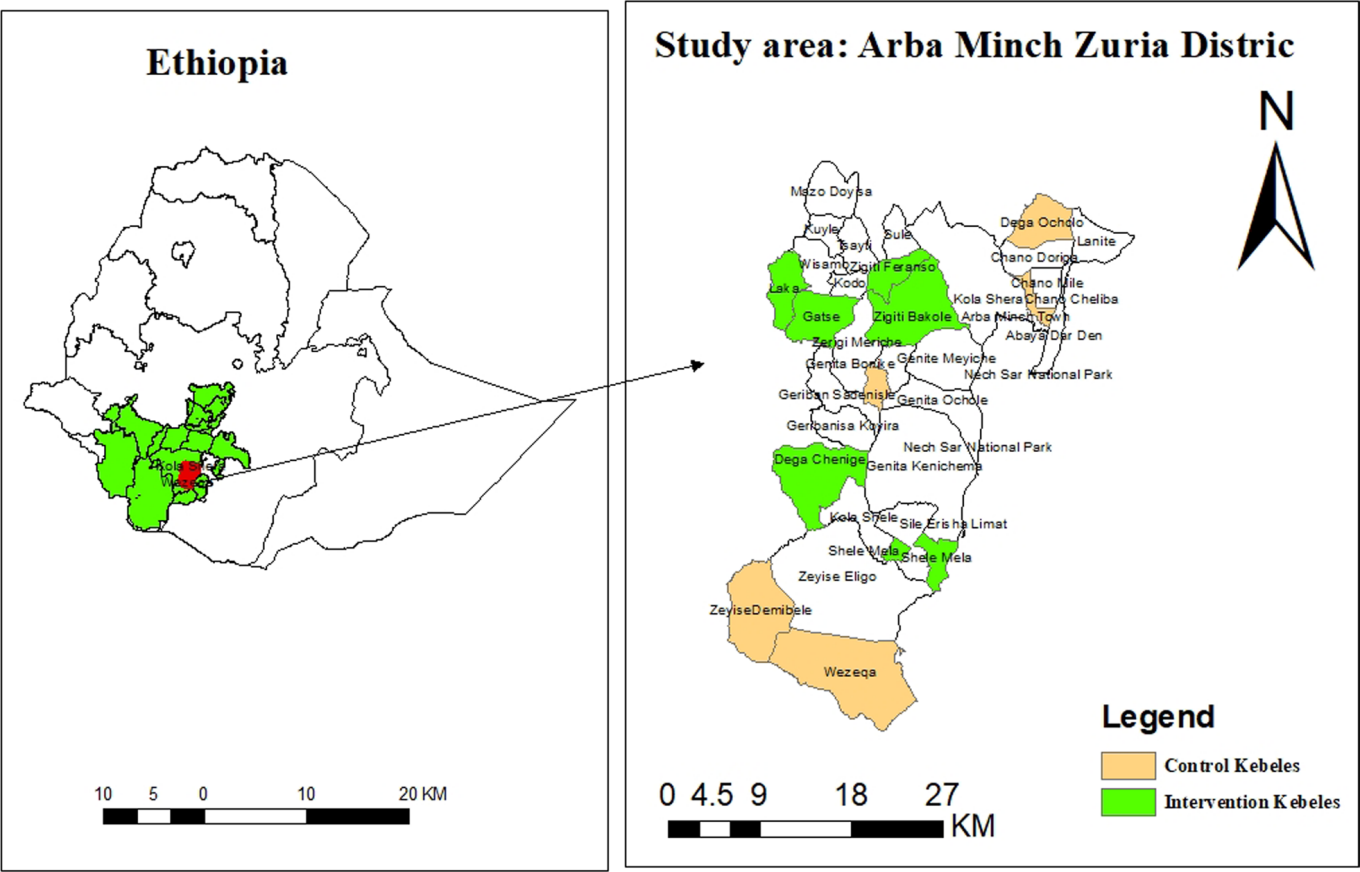
Study area

We conducted this trial at the Arba Minch HDSS site in Ethiopia from 2019 to 2021. This site has been operating in 2 districts and 10 kebeles (smallest administrative units) in total. Owing to the scattered distribution of the population coupled with the mountainous topography and the absence of proper roads, access to health services, especially EmOC facilities, was extremely limited in these districts. Additionally, the vast majority of females in the districts were uneducated and had low socioeconomic status. These districts had one of the lowest maternal health indicators in the country, with only 18.5% of births attended by skilled birth attendants [21] compared with the national average of 48% [5]. Each kebele had an average population of 3000–5000. Figure 1 shows the map of the study area. [29].

In the study area, different types of health facilities were in operation, including two public hospitals, six health centers, 32 health posts and private health facilities. At the health centres, basic EmOC is provided and when there is a need of comprehensive EmOC, the women/clients are referred to Arba Minch hospitals. Each health centre is expected to supervise five satellite health posts (the lowest community-level primary healthcare in Ethiopia) staffed with two salaried female health extension workers (HEWs) who provide promotive and preventive services for each kebele. All HEWs completed high school and one year of health extension programme training. A team of up to 30 households from the community formed the Women's Development Army (WDA), which was then divided into six smaller groups of one-to-five networks that made it easy to communicate with the community.

### Sample size and sampling method

The study's main objective was to compare the proportion of births that occur in health facilities; we used the estimated proportion of the outcome variable (births in health facilities) in the two groups. According to the 2019 EDHS report, 43% of births in rural areas were assisted by skilled providers. We assumed that the proposed intervention in this study would increase this proportion by 10 to 53%. We used Stata version 15 software to calculate the sample size for a two-sample proportions test. The following assumptions were made: alpha = 0.05 (significance level), Power = 0.80 (probability of detecting a difference between the groups), Intra-cluster correlation = 0.05 (a measure of how similar the observations are within clusters), p1 = 0.43 (estimated proportion of births in health facilities in the control group), p2 = 0.53 (estimated proportion of births in health facilities in the intervention group) and six pair of clusters.

Based on these assumptions, the required sample size was 164 pregnant women per cluster. After considering a 10% loss to follow-up, the final calculated sample size was 1082 (541 intervention clusters and 541 control clusters).

**Fig. 2:**
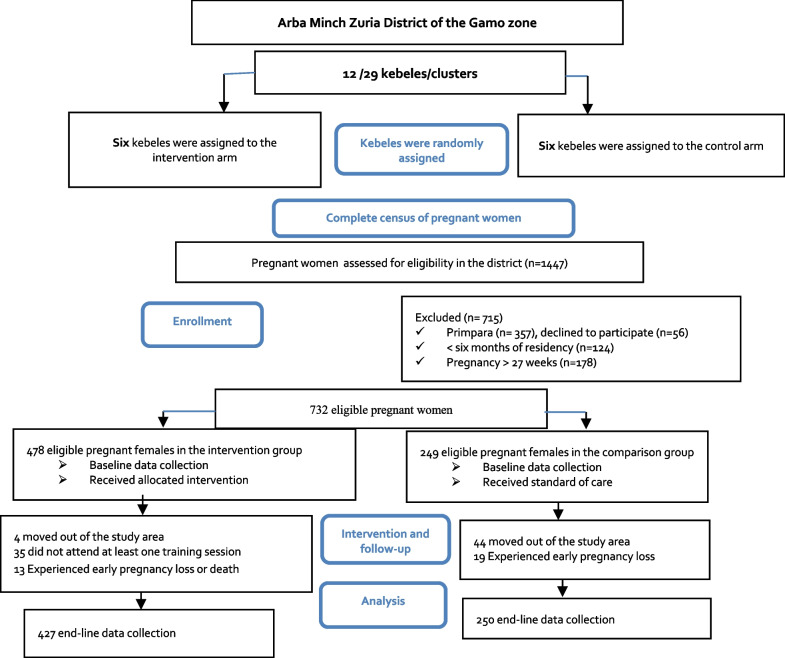
Flow diagram of the sampling procedures following the CONSORT guideline

A complete census of twelve clusters was conducted to identify pregnant women who were eligible to enrol into the study. The inclusion criteria were that the pregnant woman had resided in the selected kebele cluster for at least six months, had given birth five years preceding the survey, and had a gestational age of less than 27 weeks (end of the second trimester). Pregnant women who met the inclusion criteria but were critically ill at enrolment or reluctant to participate were excluded. A buffer cluster was formed between the clusters to prevent information from being shared between the intervention and control arms. The census identified 1447 pregnant women who underwent eligibility assessments. Of these, 732 were found to be eligible and were approached for interviews during the baseline survey. The remaining 715 did not meet the inclusion criteria or refused to participate. A flow diagram of the sampling procedures is shown in Fig. 2. The report has also followed the CONSORT statement guidelines for cluster randomized trials [30].

### Interventions and randomization

The project trained selected community members to lead educational sessions on safe motherhood topics with pregnant women. For this reason, we identified two community health workers and 15 women from each kebele cluster to train as research implementers who were literate, permanent residents in the vicinity, willing and able to volunteer, and motivated to teach other about safe motherhood practices. The implementers received two days of training from the project team from November 10–27, 2020. The training covered five sessions on danger signs during pregnancy, labour and postpartum period, birth preparedness, the importance of institutional delivery, MWHs, and timely access to EmOC.

The project staff and research implementers conducted two sessions per cluster for pregnant women in intervention clusters in 2020. The training sessions included information presentations and discussions on safe motherhood practices to increase awareness and discourage delays in seeking professional assistance. A total of 10 discussions and promotional activities, storytelling to improve their risk perception during pregnancy and childbirth were conducted with pregnant women in the community.

The training materials were developed based on the findings of formative studies as well as the WHO-developed video, "Why Did Mr. X Die, Retold?" (Available at https://iris.paho.org/handle/10665.2/43302), [20, 21, 24], and a manual on maternal and newborn health [31] The training materials were translated into the local language with WHO permission and field-tested for understandability and appropriateness in the local context.

The training approach was based on the activated health education model that engages participants in the intervention process by making them aware of positive and negative behaviours and clarifying their responsibilities [32]. We also considered the well-known three-delay model proposed by Thaddeus and Maine to improve access to EmOC in designing the intervention. The three delays are: delay in deciding to seek care, delay in reaching a health facility and delay in receiving adequate care at the health facility [33]. This model was used to determine which individual-level variables and messages were important to include in shaping maternal health behaviours, and which parts of the intervention required the most attention.

After completing training, each implementer formed a group of 15 to 20 pregnant women who lived in the same village. They held bimonthly meetings to discuss topics related to safe parenthood and provided educational materials, including a birth preparedness card that was prepared specifically for this research. The research implementers were oriented to approach pregnant women based on the local context to help them overcome barriers to using maternal healthcare services, giving birth at health centers, and seeking postnatal care.

In addition to the intervention on pregnant women, we provided a four-day training program on clinical skills in basic EmOC using the Safe Delivery application (see https://www.maternity.dk/safe-delivery-app/) to selected maternal healthcare providers in the study area. Moreover, we implemented MWH intervention in the intervention arm, which included improving MWH service to meet MWH national guidelines and creating a homelike environment for pregnant women [13]. For this reason, MWHs in the intervention clusters were provided with bedding, basic infrastructure, and food supplies. The control clusters did not receive the intervention but instead received standard care.

The unit for intervention allocation was the kebele (cluster), which refers to the smallest government administrative structure unit in Ethiopia. Since the number of kebeles may have been too small to ensure a balanced allocation between the intervention and control arms, we have matched them by dividing the 10 kebeles into 5 pairs. The pairs were matched on the basis of population, availability of maternity homes, topography and number of available midwives. As there were two kebeles in the pairs next to each other, we included both kebeles in the intervention arm to minimize the spill-over effect of information. Therefore, six kebeles were assigned to the intervention arm, and the remaining four served as the control arm. The impact of interventions on the institutional birth rate and other maternal health indicators was assessed through pre-intervention (baseline) and post-intervention (endline) surveys. Table 1 presents the baseline characteristics of the participants.

**Table 1:** Characteristics of study areas at their initial assessment in 2019

Characteristics	Intervention group	Control group
No. clusters in area (kebele)	Bakole, Pereso, Gatse, Laka, Mella and Chenge	Chano, Genta, Dembile, Ocholo and Wezeqa
No. and type of healthcare facilities in the area	One health post per cluster to serve 3000–5000 population (Six health posts) One health centre (EmOC) to serve up to 25,000 (Three health centres)	One health post per cluster to serve 3000–5000 population (4 health posts) One health centre (EmOC) to serve up to 25,000 (two health centers)
No. and type of trained health-care providers	Two HEWs at a health post Two nurses, two midwives and one health officer at a health centre	
Location and distance to the nearest hospital that can perform caesarean birth	Arba Minch Hospital, average 2 h by car	
Modes of emergency transport	The district shares one ambulance for all clusters (health centres); there is often no fuel to transfer patients to Arba Minch Hospital. In some areas, public buses are available once daily; traditional stretchers and walking are alternatives	

### Survey methodology

Following the eligibility check, we collected baseline data from 732 pregnant women in November 2020. The questionnaire gathered socio-demographic, wealth and reproductive history data. We also gathered data related to pregnancy characteristics, including the adequacy of prenatal care and details of illnesses during pregnancy, delivery and postpartum periods; types of birth attendant and, in cases of obstetric complications, referrals to district hospitals. We used the Open Data Kit (ODK, https://opendatakit.org/) smartphone application to collect the data. The questionnaire was prepared in English and translated into the local language by a person with good command of both languages. Prior to data collection, we pre-tested the tool in a cluster with similar characteristics to the sample population, and modified the logical order of the tool based on the results of the pre-test. Baseline and endline surveys were conducted by individuals who spoke the local language and had secondary school education or above, following intensive three-day training. We used the same questionnaire during the baseline and final assessments, apart from questions related to characteristics of the current pregnancy, which was only part of the baseline. During baseline and endline assessments, information about the pregnant female arm-assignment status was withheld from the data collectors. The leadership of the local health office of the intervention arm was consulted and involved from the initial site selection visit to the final assessment.

### Outcomes

The primary outcome examined in this study was delivery location (health facility vs. home). Institutional birth was used to indicate health facility delivery and skilled birth attendance, as trained health workers do not conduct deliveries outside of health facilities in these areas. Postnatal visits were also a key indicator. The questionnaire was adapted from the Ethiopia Demographic Health Survey (wealth index) [34] and the JHPIEGO birth preparedness and complication readiness monitoring tool kit for birth preparedness [35] (Table 2).

**Table 2:** Descriptions of variables and measurements for the multilevel logistic regression analysis in the Gamo Zone, southern Ethiopia, 2022

Variables	Descriptions	Measurements
*Dependent variable*		
Antenatal care	Proportion having received antenatal care during most recent pregnancy	Those attending antenatal care were coded as ‘1,’ ‘0’ otherwise
Institutional birth	Proportion giving birth in a health facility	Those giving birth in a health facility were coded as ‘1,’ ‘0’ otherwise
Postnatal care	Proportion receiving postnatal care after delivery	Those receiving postnatal care were coded as ‘1,’ ‘0’ otherwise
*Level 2 (higher level) independent variable*		*Communal (cluster level) variable*
Climatic zone	The usual place of residence	The three common agro-ecological zones: Highland, Midland and Lowland
*Level-1 (lower-level) independent variables*		*Individual level variables*
Woman’s age	Age at interview (completed years)	Absolute continuous numbers
Wealth quintiles	Household assets (e.g. home, land and livestock); wealth index was computed using principal component analysis	Wealth status was categorized and ranked from poorest to wealthiest in quintiles
Education	Highest level of education attained	Ordinal variable based on the highest level of education attained (i.e. no formal education, primary education and secondary or higher) categorized into formal school attendance (lowest to highest) or no formal school attendance
Mother’s occupation	Proportion working to earn money in addition to household chores	Occupation categorized as ‘earn money’ or ‘housewife’
Religion	Religious background	Each religion was entered and later recoded as ‘Orthodox Christian,’ ‘Protestant’ or ‘Other.’ ‘Other’ included choices they were too sparse for logistic regression
Number of children	Number of children in household	Absolute numbers and letters categorized as ‘1 child’ or ‘ ≥ 2 children ‘
Awareness of danger signs	Proportion that knew the danger signs of pregnancy, childbirth and postpartum period	Those who successfully listed three danger signs were categorized as ‘good awareness,’ ‘having poor awareness’ otherwise
Awareness of birth preparedness	Proportion that understood birth preparedness	Those who mentioned three of the birth-preparedness components were categorized as ‘good awareness,’ ‘having poor awareness’ otherwise
Practice birth preparedness	Proportion that made prior arrangements for birth and complications	Those who made at least three basic birth preparedness steps were categorized as ‘well prepared,’ ‘less prepared’ otherwise

The secondary outcomes examined in this report include knowledge of obstetric danger signs and birth preparedness practices. Knowledge of obstetric danger signs was measured by asking pregnant women: "Do you know any danger signs for a woman during pregnancy, who has begun labor and delivery, or after delivery? If yes, which danger signs does she know?" The pregnant woman's unprompted responses were recorded. Danger signs and their explanations were listed on the questionnaire. Adequate knowledge was defined as the ability to recall three or more obstetric danger signs during pregnancy, delivery, and the postpartum period.

Birth preparedness was measured by coding the pregnant women's unprompted responses to the questions, "Did you make any plans or preparations for the delivery and potential complications before your most recent birth? If yes, what type of preparations did she make?" Identifying the nearest health facility where one would go if there were problems, arranging transportation to the health facility, setting aside money to pay for transportation and medical care, talking with the nearest trained health worker about the upcoming birth, deciding who would assist with the birth, and planning to stay at an MWH. Adequate knowledge/preparation was defined as the ability to recall or prepare three or more birth preparedness activities and having made three or more birth preparations [35].

### Data analysis

We exported the ODK questionnaire data into a comma-separated variable format and then imported it into Stata v.17.0, a statistical analysis software tool (StataCorp, College Station, TX, USA). We used Chi-square and t-tests to compare the characteristics of the participants in the two study arms at the baseline and final assessment.

To evaluate changes in knowledge about danger signs, birth preparedness, and service utilisation, we used McNemar's test. To further examine the change in institutional delivery service care utilization (home vs. health facility) in the intervention arm compared to the change in the control arm, we used generalised mixed-effects logistic regression models with a random intercept at the kebeles to estimate the effectiveness of the intervention on the outcomes. The following independent variables were used to estimate the effectiveness: intervention status, educational status, agro-ecological zone, baseline findings of outcome variables and socioeconomic status. All hypothesis tests were two-sided, with the level of statistical significance set to p < 0.05.

## Results

### Socio-demographic characteristics of the participants

The socio-demographic characteristics of participants interviewed in the intervention and control arms are summarized in Table 3. At the baseline assessment, intervention and control arm participants were similar in terms of their age distribution, number of living children, and husband's occupation. However, the intervention arm had a significantly lower education level, and most participants belonged to the first and second socioeconomic groups residing in the highland area. Given these differences, we adjusted for education, socioeconomic group, and agroecological zone in the final analysis.

**Table 3:** Sociodemographic characteristics of participants interviewed in intervention and control clusters at baseline and final assessments in the Gamo Zone, southern Ethiopia, 2022

Variables	Baseline (727)		Final (617)	
	Intervention (478)	Comparison (249)	Intervention (427)	Comparison (190)
*Continuous variables*				
Mean age (SD)	28.5 (4.7)	28.0 (3.1)*	28.9 (4.8)	28.9 (3.4)*
Mean age of husband (SD)	32.6 (5.5)	32.5 (4.2) *	33.5 (5.4)	34.3 (4.6)*
*Categorical variables*				
Gamo ethnic group, n (%)	457 (95.8)	101 (40.4)	409 (95.7)	83 (43.6)
Protestant, n (%)	333 (69.8)	210 (84)	316 (74)	158 (83)
Some formal education, n (%)	120 (25.1)	103 (41.2)	88 (20.6)	75 (39.4)
First and second quantile household index, n (%)	235 (49.3)	56 (22.4)	223 (52.2)	24 (12.6)
Currently living in highland area, n (%)	277 (58.1)	33 (13.2)	236 (55.3)	25 (13.2)
Has a job that earns money, n (%)	23 (4.8)	42 (16)	9 (2.1)	30 (15.7)
Husband farming, n (%)	381 (79.8)	195 (78)*	360 (84)	138 (72.6)
Husband with some formal education, n (%)*	120 (25.2)	103 (41.2)	115 (26.9)	84 (44.2)
Two or more living children, n (%)	272 (57)	133 (53.2)*	427 (100)	190 (100)

^*^p-value from chi-square (categorical variables) or *t*-test (continuous variables) comparing the intervention and comparison groups (*p* < 0.05)

### Knowledge of obstetric danger signs

The proportion of participants aware of at least three danger signs during pregnancy increased from 48.6% at the baseline to 62.3% at the endline in the intervention arm. However, there was no change in the control arm (48.4% baseline vs. 44.7% endline) (see Table 4). The change in the proportion of awareness of three or more danger signs during pregnancy after intervention was statistically significant (p < 0.01).

**Table 4:** Changes in knowledge of danger signs and birth preparedness following intervention in the Gamo Zone, southern Ethiopia 2022

Indicators	Intervention arm		Control arm		McNemar’s Test
	Baseline (478)	End-line (427)	Baseline (249)	End-line (190)	OR (95% CI)
No. (%) of participants who knew three pregnancy danger signs^a^	232 (48.6)	266 (62.3)	121 (48.4)	85 (44.7)	1.5 (1.1–2.2) *
No. (%) of participants who knew three childbirth danger signs^a^	221 (46.3)	273 (63.9)	112 (44.8)	95 (50)	2.5 (1.7–3.7) *
No. (%) of participants who knew three postnatal danger signs^a^	215 (45.1)	271 (63.5)	71 (28.4)	69 (36.3)	4.7 (2.9–8.1) *
No. (%) of participants who knew > = 3 components of BPCR^b^	188 (39.4)	273 (63.9)	150 (60.0)	108 (56.8)	2.5 (1.8–3.6)*
No. (%) of participants who practised > = 3 components of BPCR^b^	172 (36.1)	293 (68.6)	83 (33.2)	74 (38.9)	6.7 (4.3–11)*
MWH awareness	262 (54)	379 (88.79)	53 (21.29)	58 (30.5)	5 (3.5–7.3)*
Institutional birth rate ^c^	174 (36.4)	224 (52.5)	164 (65.6)	105 (55.3)	1.5 (1.1–2) *
Postnatal visits	158 (33.1)	142 (33.3)	81 (32.4)	70 (36.8)	1.1 (0.4–3.1)

^*^Statistically significant at *p* < 0.05

^a^Adequate knowledge was defined as being able to recall three or more obstetric danger signs during pregnancy, delivery and childbirth

^b^Adequate knowledge was defined as being able to recall three or more birth preparedness and complication readiness (BPCR) components

^c^Location of delivery (home vs. health facility); health facility was used as an indicator of institutional birth rate as trained health workers do not conduct deliveries outside the health facility in these areas

Similarly, the proportion of participants aware of three or more childbirth danger signs increased from 46.3% at the baseline to 63.9% at the endline. The change was statistically significant (p < 0.001). Regarding awareness level and the use of MWHs, there was a significant difference in the percentage of participants who knew the function and location of MWHs from baseline to endline. However, there was no significant difference in MWH use.

### Birth preparedness practices

As summarized in Table 4, participants who were aware of three or more basic aspects of birth preparedness increased from 39.4% at the baseline to 63.9% at the end of the study, and the change was statistically significant (*p* < 0.001). Similarly, there was a statistically significant change in the proportion of mothers who prepared for at least three of the basic criteria of birth preparedness among participants following intervention (*p* < 0.001).

### Institutional delivery care and postnatal care visits

The proportion of participants who gave birth in healthcare facilities increased significantly in the intervention arm, from 36.4% at baseline to 52.5% at endline. This change was statistically significant (p = 0.01). After adjusting for background differences, a mixed-effects generalized linear regression analysis showed a significant association between intervention and institutional delivery care utilization (see Table 5). The AOR of giving birth in a healthcare facility for participants in the intervention arm was 2.8 (95% CI: 1.2–6.4), meaning they were 2.8 times more likely to give birth in a healthcare facility than those in the control arm. The goodness of fit of the models for the three-level analyses is presented in Table 6. The interventions did not result in a significant change in postnatal care visits.

**Table 5:** Generalized mixed-effects multilevel logistic regression of the effect of the intervention on institutional birth rate among pregnant women in the Gamo Zone, southern Ethiopia 2022

		Institutional birth rate		
		Model 1 (level-2 variables) AOR (95% CI)	Model 2 (level-1 variables) AOR (95% CI)	Model 3 (full model) AOR (95% CI)
*Level 2: higher level variable*				
Climatic zone (Highland ref.)	Lowland	2.3 (3.1, 3.3)	–	1.7 (1.2, 2.6)*
*Individual-level variables*				
Intervention status	Experimental group	–	1.8 (0.8, 4.3)	2.8(1.2, 6.4)*
Mother’s level of education (no formal schooling ref.)	Formal education	–	0.8 (0.5, 1.5)	0.9 (0.5, 1.6)
Wealth index (first quantile ref.)	Fifth quantile	–	1.4 (1.2, 1.7)	1.3 (1.1, 1.6)*
End-line BPCR practice (less prepared ref.)	Well-prepared	–	1.9 (1.2, 2.9)	1.8 (1.2, 2.8)*
End-line ANC (no ANC visit ref.)	Yes	–	1.4 (0.8, 2.4)	1.4 (0.8, 2.3)
Baseline Institutional birth (homebirth ref.)	Health facility	–	2.5 (1.4, 3.4)	2.1 (1.4, 3.2)*

(a) estimated using *meglm family* (Bernoulli) link (logit) command in Stata. * *p* < 0.05)

(i) Dependent variables: Institutional birth rate; (ii) cluster variable: kebele (10); (iii) model 0: empty model; model 1: cluster-level variable included; model 2: individual level variables included; model 3: full model (all cluster- and individual-level variables included)

**Table 6:** Goodness-of-fit of the generalised mixed-effects multilevel logistic regression model for the outcome variable

Institutional birth rate	Random effects as level-2 variance	Rho–intra-class correlation	Log likelihood (LR) deviance	Wald χ2	Significance of LR test vs. logistic regression (*p* value)
Model 0	0.56	15%	60.43	–	< 0.001
Model 1	0.35	9.6%	32.04	16.33	< 0.001
Model 2	0.28	7.8%	14.6	48.57	< 0.001
Model 3	0.22	6.2%	9.33	53.21	0.0011

## Discussion

The trial evaluated the effectiveness of a set of community-based interventions to reduce delays in accessing skilled care in rural Gamo zone. Accordingly, the results showed that the interventions were effective in improving women's knowledge of obstetric danger signs, birth preparedness practices, and use of skilled care services in the study area. Our findings are consistent with the findings of several community-based interventions conducted in LICs [36–40].

Despite efforts to address the barriers to accessing skilled care, only a small number of rural women give birth in health facilities in rural Ethiopia [5]. A previous study from rural Gamo zone revealed that the three maternal delays were all high, with the first delay being the most common [23]. The study also found that half of the pregnant women experienced delays in seeking medical care for obstetric emergencies due to a lack of knowledge of obstetric danger signs, lack of birth preparedness, negative perceptions of facility care, and geographic barriers [23].

Poor awareness of health and obstetric danger signs was common in rural communities where only 30% of women received formal education [24]. Other studies have also shown links between women education and maternal health outcomes and maternal healthcare utilization, including when to initiate antenatal and postnatal care [23, 24]. Our study found that the intervention had a significant positive effect on awareness about obstetric danger signs and birth preparation in the intervention group. However, the control group also showed some improvements during the study period. The control group may have improved due to their relatively higher educational and socioeconomic status, information contamination from the intervention arm, or the Hawthorne effect [41]. However, the intervention arm still had a greater increase in awareness of obstetric danger signs, 17.6% compared to the control arm, which increased 5.2% from the baseline. Besides the routine health information dissemination, providing pregnant women with tailored, evidence-based information supported by video can increase their knowledge and lead to behavioural changes, such as seeking care on time.

Improved awareness alone cannot lead to increased utilization of existing services, especially skilled care utilization, which is significantly affected by financial, logistical, and sociocultural and perception about the quality of care related barriers [24]. The three-delay framework we used to set up our study identifies the first delay in decision-making to seek skilled care and can be attributed to a lack of awareness, but a lack of trust and poor quality of care in the health system can also contribute to it [33].

We found that the proportion of women who prepared for childbirth increased significantly (by 32.5%), as did the proportion of women who gave birth in healthcare facilities (by 16.1%). This finding highlights the gaps in routine care and the importance of integrated community-based packages of interventions for improving maternal health. However, it also suggests that not all women who are prepared for childbirth will actually give birth in healthcare facilities or receive postnatal care. Therefore, actions aimed at creating demand for existing services should be accompanied by community-level efforts to follow up on pregnant women to ensure they implement the message and create supportive environments that promote positive behaviour [31, 39, 42].

We implemented MWHs as part of our interventions to address the second delay in the study area and improved the MWHs in the intervention arm to make them comfortable places for women to stay during the last few weeks of their pregnancy. Although we found that a higher proportion of women in the intervention arm were aware of MWHs, there was no significant difference in staying at MWHs between the two arms. Thus, in addition to addressing the awareness gap, it is important to consider barriers to stay MWHs, such as the support system at the family level, the willingness of husbands to allow their wives to stay in MWHs and cultural restrictions [22]. The insignificant changes in staying at MWHs may also be attributed to the COVID-19 pandemic, which disrupted access to and utilization of maternal and child health services [43]. COVID-19-related movement restrictions, transportation challenges, and anxieties about overexposure may have acted as barriers for women who wanted to stay at MWHs and giving birth at healthcare facilities. According to one study, the pandemic affected not only those who would like to stay at MWHs but also the institutional delivery proportion, which decreased by up to 45% [44]. Therefore, the change in institutional delivery care utilization in the intervention arm was more likely due to the intervention, which created a better understanding of possible problems with home births. Furthermore, even after taking into account other factors that could have influenced the results, such as women's education status, socioeconomic status, and agroecological zone, the intervention was still found to increase institutional delivery care utilization significantly.

Through its HEP, the Ethiopian government has recognized that community-based interventions are essential to improving maternal health. There is strong evidence that the HEP has achieved much regarding maternal health, especially in rural areas [45, 46]. However, a study found that HEWs had poor knowledge of pregnancy complications despite being trained in maternal health [47]. Their poor knowledge makes identifying and referring women needing urgent care challenging. Another study has also reported that the HEP had improved maternal and newborn healthcare practices but had little effect on institutional and skilled deliveries [48]. In our study, HEWs received video-supported training on timely referral, the consequences of delay, and linking pregnant women to MWHs in the intervention arm, which could contribute to improving institution delivery car utilization. We recommend using innovative teaching methods, such as video-supported education, to improve HEWs' awareness of maternal health and enhance community engagement. We also recommend conducting further research into the effectiveness of various training interventions, such as video-assisted education, on a large scale.

The primary aim of our study was to increase demand among pregnant women and their male partners, as this can lead to better and more sustainable changes. However, since most men were farmers and the intervention occurred during the harvest season, only pregnant women attended the intervention sessions. Women also emphasized the importance of including men, who are typically the decision-makers regarding healthcare services. Several studies have shown that males with knowledge of pregnancy and delivery complications are more likely to advocate for professional care [49, 50]. In countries like Ethiopia, male family members make most decisions regarding pregnancy and childbirth, including the place of delivery; therefore, well-informed males should contribute to positive decision-making for females. Therefore, future research and interventions should focus on including husbands, rather than only pregnant women, for the intervention to be successful and sustainable.

### Strengths and limitations of the study

To the best of our knowledge, this is the first study in rural Gamo zone, southern Ethiopia to evaluate the impact of a package of community-based interventions on institutional delivery. However, the study had some limitations. First, the reliance on participant reports could have introduced social desirability bias. Second, although the clusters were randomly assigned to the intervention and control arms, there was cluster-level variability in background characteristics. These differences could have resulted in control arm participants having better access to information and resources than those in the intervention arm. Nevertheless, it is remarkable that the intervention arm achieved awareness of obstetric danger signs, birth preparedness practices, and selection of health facilities for childbirth comparable to or even higher than the control arm.

## Conclusions

This study has demonstrated that an integrated community-based intervention package can increase awareness and utilization of maternal health services, narrow the inequity between the urban and rural areas. The findings of this study suggest that stimulating demand for existing services can improve institutional delivery care utilization, even during pandemics. We recommend incorporating audio-visual education that starts during pregnancy and continues postpartum and improving MWH services into routine maternal healthcare services in Ethiopia.

Overall, inequities in skilled care utilization are a complex issue that cannot be solved by the health system alone, and the social determinants of health, such as poverty, gender inequality, and a lack of education, also play a role. Therefore, it is crucial to take a multi-sectoral approach and work with various stakeholders to address the root causes of inequities in access to care. Research is also needed to evaluate multi-pronged interventions' effectiveness and identify the best way to improve access to skilled care services for all women.

## Data Availability

The data supporting this study's findings are available from Arba Minch University. There are some restrictions to the availability of these data due to license, so the data are not publicly available. However, the data may be made available from the authors upon reasonable request and with the permission of Arba Minch University.
